# Molecular and MALDI-TOF MS identification of swallow bugs *Cimex hirundinis* (Heteroptera: Cimicidae) and endosymbionts in France

**DOI:** 10.1186/s13071-021-05073-x

**Published:** 2021-11-27

**Authors:** Fatima Zohra Hamlili, Jean-Michel Bérenger, Adama Zan Diarra, Philippe Parola

**Affiliations:** 1 IHU-Méditerranée Infection, Marseille, France; 2 Aix Marseille Univ, IRD, AP-HM, SSA, VITROME, Marseille, France

**Keywords:** *Cimex hirundinis*, MALDI-TOF MS, Swallow bug, *Wolbachia*, *Wolbachia massiliensis*, France

## Abstract

**Background:**

The Cimicidae are obligatory blood-feeding ectoparasites of medical and veterinary importance. We aim in the current study to assess the ability of MALDI-TOF MS to identify *Cimex hirundinis* swallow bugs collected in house martin nests.

**Methods:**

Swallow bugs were picked out from abandoned nests of house martin swallows and identified morphologically to the species level. The bugs were randomly selected, dissected and then subjected to MALDI-TOF MS and molecular analyses.

**Results:**

A total of 65 adults and 50 nymphs were used in the attempt to determine whether this tool could identify the bug species and discriminate their developmental stages. Five adults and four nymphs of *C. hirundinis* specimens were molecularly identified to update our MS homemade arthropod database. BLAST analysis of *COI* gene sequences from these *C. hirundinis* revealed 98.66–99.12% identity with the corresponding sequences of *C. hirundinis* of the GenBank. The blind test against the database supplemented with MS reference spectra showed 100% (57/57) *C. hirundinis* adults and 100% (46/46) *C. hirundinis* nymphs were reliably identified and in agreement with morphological identification with logarithmic score values between 1.922 and 2.665. Ninety-nine percent of *C. hirundinis* specimens tested were positive for *Wolbachia* spp. The sequencing results revealed that they were identical to *Wolbachia massiliensis*, belonging to the new T-supergroup strain and previously isolated from *C. hemipterus*.

**Conclusions:**

We report for the first time to our knowledge a case of human infestation by swallow bugs (*C. hirundinis*) in France. We also show the usefulness of MALDI-TOF MS in the rapid identification of *C. hirundinis* specimens and nymphs with minimal sample requirements. We phylogenetically characterized the novel *Wolbachia* strain (*W. massiliensis*) infecting *C. hirundinis* and compared it to other recognized *Wolbachia* clades.

**Graphical Abstract:**

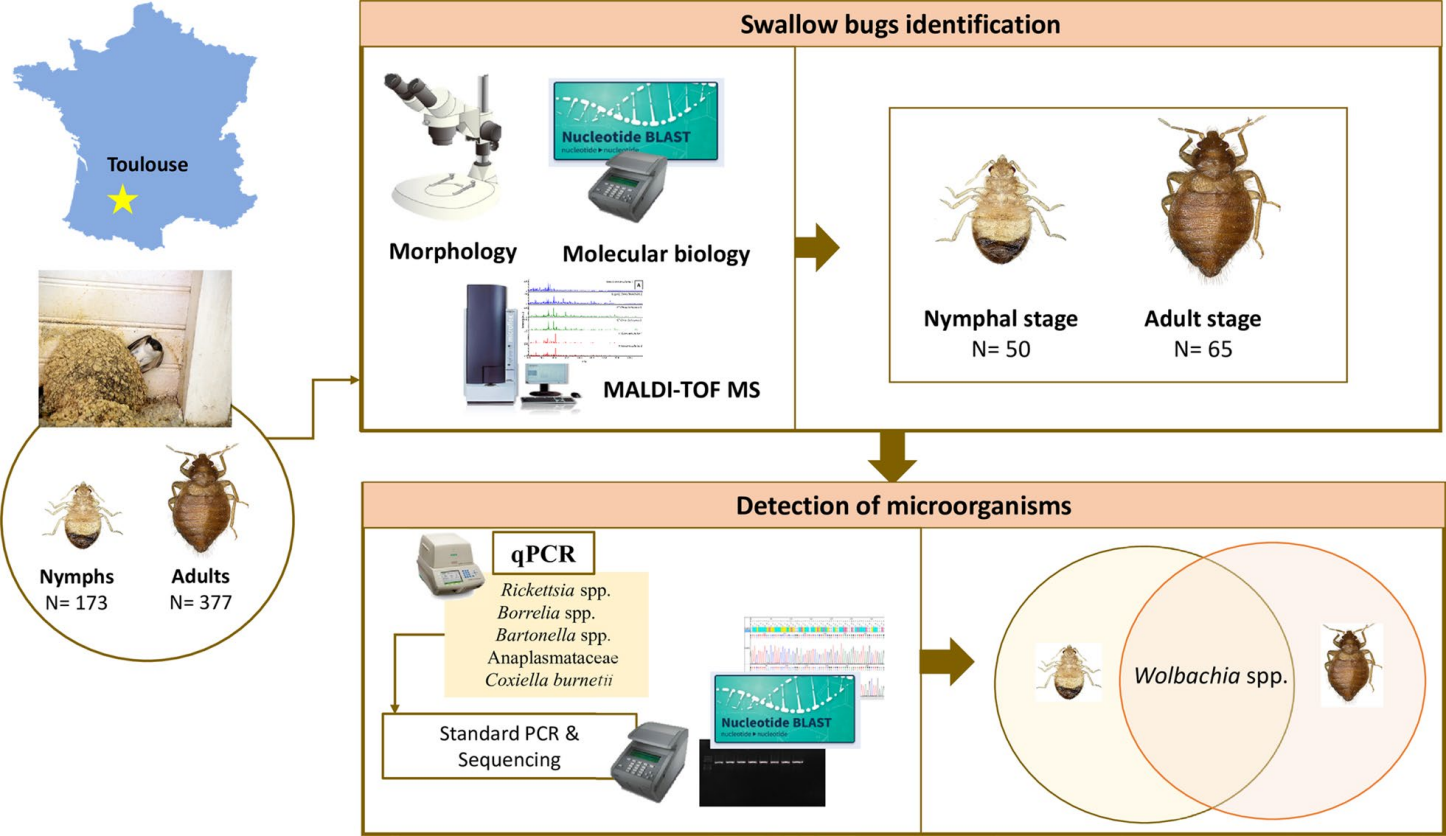

**Supplementary Information:**

The online version contains supplementary material available at 10.1186/s13071-021-05073-x.

## Background

The “true bugs” refer to the order Hemiptera, with > 42,000 species in 90 families worldwide [1, 2]. This order comprises insects, including predatory entomophagous insects that feed on insects and small invertebrates, phytophagous insects and three families that are strictly hematophagous [3]. The Cimicidae family includes around 100 species grouped into 24 genera [4]. This family can be differentiated from other Hemiptera by being flat in shape, ovoid, flightless and wingless [3, 5].

In Europe, the Cimicinae subfamily is the only one prevalent. It is represented by the genus *Cimex*, which includes seven species [6, 7]. Within the *Cimex* group, two cosmopolitan species, *C. lectularius*, the common bed bug, and *C. hemipterus*, the tropical bed bug, feed on human blood [8–10]. Otherwise, *Cimex columbarius*, *C. pipistrelli* and *C. dissimilis* occasionally feed on human blood when their preferred hosts (bats and birds) are absent [6, 11, 12]. Three common species are involved in swallow bug infestation: the North American swallow bug *C. vicarius*, which is an ectoparasite of the cliff swallow, rarely reported in the barn swallow and house sparrow [13–16]; *Cimex hirundinis*, which is found in Eurasia, exclusively common to house martin nests and other birds; *Cimex montandoni*, which is found specifically in Romania in sand martin nests [7, 17]. *Cimex vicarius* is the only known vector of Buggy Creek virus (BCRV; Togaviridae, Alphavirus), which causes western equine encephalitis [18, 19]. Another arbovirus, the strain responsible for Venezuelan equine encephalitis (Tonate virus), has also been isolated in *C. vicarius* [20].

Under certain conditions, *C. hirundinis* are able to feed on human blood, and their bite is known to be more painful than that of bedbugs [6, 7, 21, 22]. However, except for experienced entomologists, it is challenging to make a morphological distinction between *Cimex* spp. and even other arthropods. In addition, the number of entomologists is declining, and adapted documentation is sometimes not accessible [23]. The molecular approach has been assessed for its potential to overcome these limitations. Conversely, the molecular tool is relatively laborious, requires high-cost reagents and depends on both the availability of high-quality reference sequences in the GenBank database and the use of the correct gene fragment [23–25].

Over the past decade, the matrix-assisted laser desorption/ionization time-of-flight mass spectrometry (MALDI-TOF MS) technique has widely revolutionized the clinical microbiology field [26]. It has also emerged in medical entomology. MALDI-TOF MS has been shown to be rapid, reliable and notably inexpensive (as soon as the device is available) for identifying various species of arthropods [27]. Recently, Benkacimi et al. [25] showed that this innovative tool could be used as an alternative method to identify and discriminate between *C. hemipterus* and *C. lectularius*.

Our study aimed to assess the ability of MALDI-TOF MS to identify swallow bugs collected from abandoned house martin nests in France. Molecular tools were also used to identify these swallow bugs and screen them for carriage of microorganisms.

## Methods

### Swallow bugs sampling and morphological identification

**Fig. 1:**
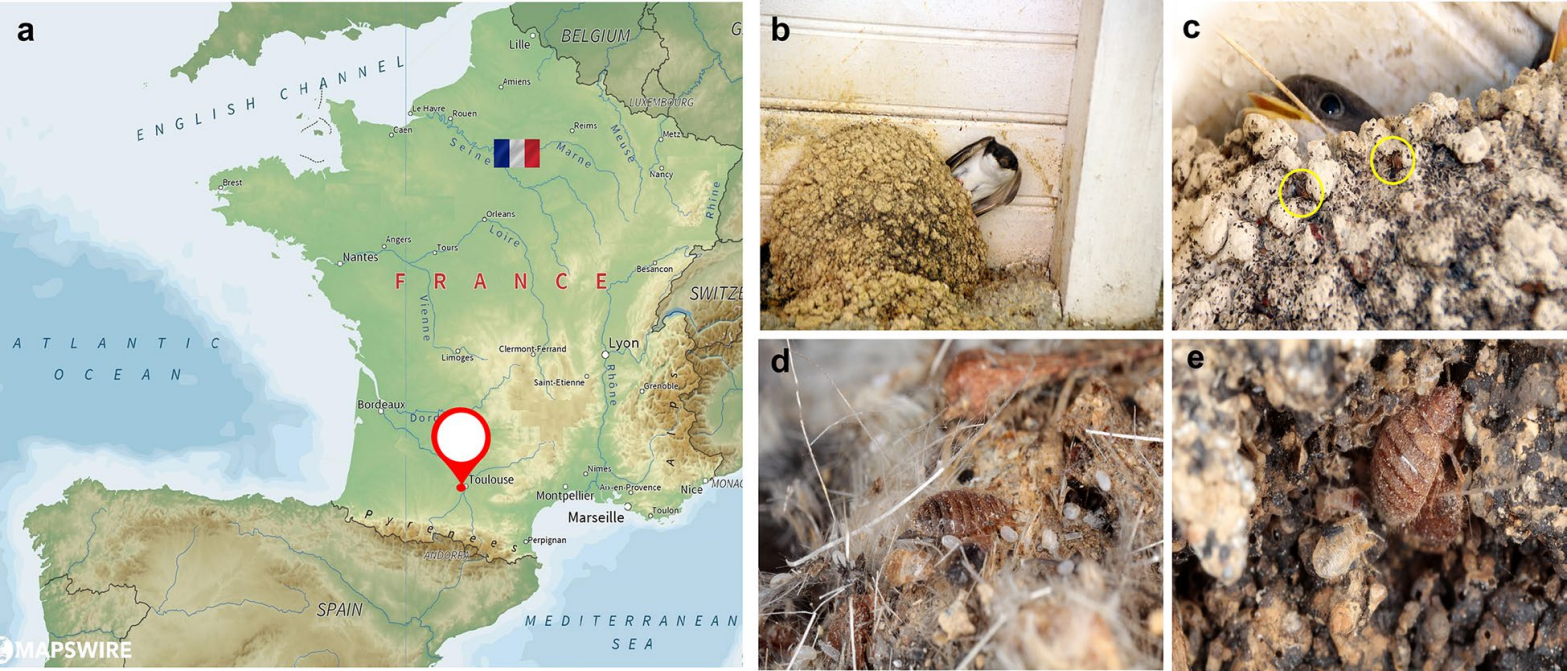
Map of France showing the sample collection site. **a** Toulouse, indicated with a red checkpoint logo. **b**, **c** Infestation of martin house nests by swallow bugs (*C. hirundinis*) built under the house eaves. **d**, **e** Presence of *C. hirundinis* adults, nymphs and eggs. Source of the map of France https://mapswire.com/countries/france/

Five hundred swallow bugs were sampled from abandoned swallow nests in a house located in Toulouse (43°36′16″N, 1°26′38″E) in southwest France in July 2020 (Fig. 1a). The house martin swallows [*Delichon urbicum* (Passeriformes, Hirundinidae)] built jug-shaped mud nests under the eaves of the house, represented in Fig. 1b. The sampling was conducted in highly infested nests (Fig. 1c–e). Abandoned nests were placed in plastic storage containers, carefully transported to the insectarium of Marseille and broken into small pieces to pick out the swallow bugs. The swallow bugs were harvested using forceps, counted and then stored at − 20 °C. The morphological identification to the species level was assessed and confirmed by an expert entomologist (JMB) using the identification keys [6, 7]. A VHX-7000 digital microscope (Kayence, Osaka, Japan) and electron microscope (SEM Hitachi TM4000 Plus) were used to photograph morphological details. For the analysis, the insect stage and species were codified on the tube.

### DNA extraction and molecular identification of swallow bugs

DNA extraction was performed from the half body of each specimen using an EZ1 DNA Tissue Kit (Qiagen) following the same DNA extraction protocol as described by Benkacimi et al. [25]. The swallow bug specimens (nymphs and adults) added into the MS reference database were subjected to standard PCR in an automated DNA thermal cycler (Applied Biosystems, 2720, Foster City, CA, USA) using Folmer’s universal *COI* (cytochrome oxidase subunit I) barcoding primers (LCO1490, HCO2198) targeting 710 base pairs [28]. The thermocycler program used for the amplification of the *COI* was previously described by Benkacimi et al. [25]. The sequences obtained were used to perform BLAST searches [29] via the National Center for Biotechnology Information (NCBI) GenBank sequence and were then aligned using MEGA7 [30]. A phylogenetic tree was constructed and edited using the maximum likelihood method with model selection determined by MEGA7 and Figtree 1.4.2, respectively [30–32]. Statistical support for internal branches of the trees was evaluated by bootstrapping with 500 iterations.

### MALDITOF MS sample preparation for analysis

Specimens of swallow bugs (*n* = 115) were rinsed successively in 70% ethanol followed by two baths of distilled water and were dried on sterile filter paper [25]. The heads of adults (*n* = 65) and the cephalothoraces (head and thorax) of nymphs (*n* = 50) were dissected under a Leica ES2 stereomicroscope 10×/30× using a new sterile blade. They were then immersed for 2.5 min in distilled water, rinsed with distilled water and immersed for 2 min in 200 μl of 70% formic acid and 200 μl of 50% acetonitrile. The dissected parts were dried on sterile filter paper for MALDI-TOF MS analysis [25]. The remaining body parts were conserved at − 20 °C for molecular biology and supplementary analysis. The cephalothoraces were homogenized in 15 μl and the heads in 40 μl of the extraction solution (70% formic acid and 50% acetonitrile) using glass beads (1.0 mm diameter, BioSpec Products). All preparations were homogenized using the TissueLyser instrument (Qiagen, Germany). One microliter of the supernatant of the protein extract from each sample was spotted in quadruplicate on a MALDI-TOF MS steel target plate (Bruker Daltonics, Germany). The spots were left to dry and then covered with 1 μl of matrix solution composed of saturated *α*-cyano-4-hydroxycynnamic acid (Sigma, Lyon. France), 50% acetonitrile, 2.5% trifluoroacetic acid and HPLC-grade water [33]. The target plate was dried at room temperature before being inserted into the MALDI-TOF MS instrument (Bruker Daltonics, Germany) for analysis.

### MALDI-TOF MS parameters

Protein mass profiles were generated using a Microflex MALDI-TOF mass spectrometer (Bruker Daltonics) with Flex Control software (Bruker Daltonics), with parameters previously described [34]. The profiles of the spectra obtained were viewed using Flex Analysis, version 3.3, and MALDI Biotyper, version 3.0, software and ClinProTools v.2.2 for data processing.

### MALDI-TOF MS analysis and reference database creation

The reproducibility of the MS spectra generated from adult and nymph swallow bugs was visualized with Flex analysis v.3.3 and then exported to ClinProTools v.2.2 software packages (Bruker Daltonics, Germany) for data processing (smoothing, baseline subtraction) [25]. Intra-species reproducibility and inter-species specificity were assessed by comparing and analyzing the spectral profiles obtained from the four spots of each individual specimen. Spectra of bad quality were excluded from the analysis [< 3000 arbitrary units (a.u.)]. An MS dendrogram was created using MALDI-Biotyper software v.3.0 to visualize the heterogeneity level of MS spectra from adult and nymph swallow bugs (hierarchical clustering of the mass spectra). Good-quality spectra (high peak intensity and reproducibility) were then added to our MALDI-TOF MS in-house database after being molecularly confirmed.

### Blind tests

The blind test was performed (MALDI-Biotyper software v.3.0, Bruker Daltonics) using swallow bug specimens, with the exception of those used as MS reference spectra. The accuracy of species identification was evaluated using obtained log-score values (LSVs). This value can range from 0 to 3. The spectrum with the highest log score value [LSV] among the four spots was selected as a valid identification [33].

### Microorganism detection in swallow bugs

DNA from 115 swallow bugs, including 65 adults and 50 nymphs, was screened by qPCR using primers and probes targeting specific sequences of the following bacterial pathogens: *Rickettsia* spp. (*RKND03*), *Borrelia* spp. (*Bor 16S*), *Bartonella* spp. (*Barto ITS2*) and *Coxiella burnetii* (*IS30A*), *Anaplasmataceae* spp. (*23S rRNA*) [33]. Positive samples for *Anaplasmataceae* spp. were then submitted to the qPCR system specific for detecting *Wolbachia* spp. [35]. For each qPCR plate, negative (qPCR reaction mix without DNA) and positive (DNA from our laboratory cultures) controls were used. Four *Wolbachia*-positive samples were submitted to standard PCR targeting 438 base pair fragments of *16S rRNA* [36] and targeting 560 base pair fragments of the *ftsZ* gene [37] (Table 1) and sequencing to identify *Wolbachia* species. Phylogenetic analyses based on *16S rRNA* and *ftsZ* gene sequences were performed using the maximum likelihood method and the model selected by MEGA7 [30, 31, 38]. Statistical support for internal branches of the trees was assessed by bootstrapping with 1000 iterations (only bootstrap values ≥ 50 were retained).

**Table 1:** Primers used for *Wolbachia* sequencing

Microorganism	Target gene	Primer	Sequence (5′–3′)	Tm (°C)	Amplicon size (bp)	References
*Wolbachia* spp.	*16S rRNA*	W-Specf W-Specr	CATACCTATTCGAAGGGATAG AGCTTCGAGTGAAACCAATTC	60	438	[36]
	*ftsZ*	Wol.ftsZ.363.f Wol.ftsZ.958.r	GGRATGGGTGGTGGYACTGG GCATCAACCTCAAAYARAGTCAT	59.50	560	[37]

## Results

### Morphological characterization

**Fig. 2:**
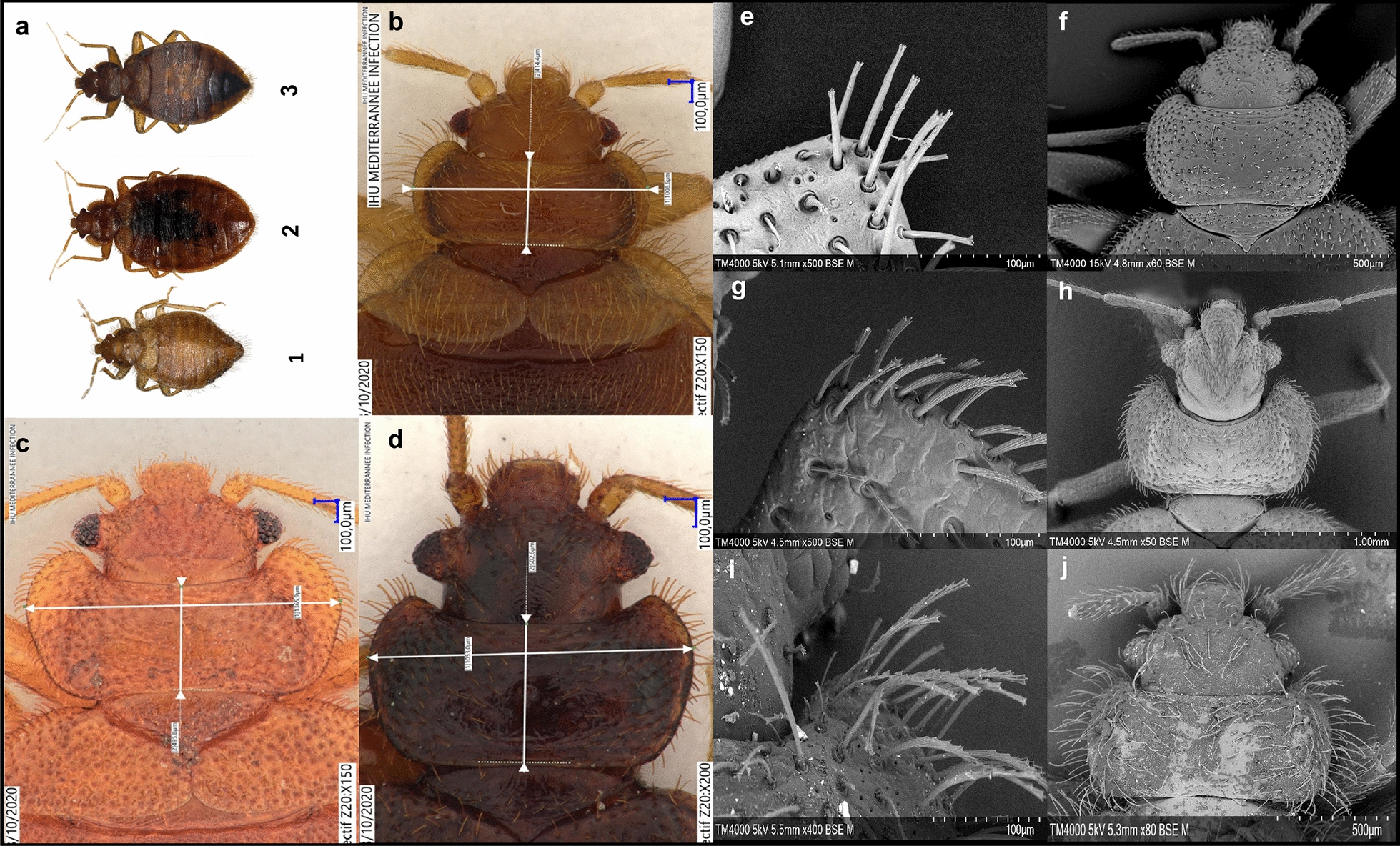
Digital microscope (DM) and scanning electron microscope images (SEM) of a swallow bug and laboratory-reared bed bugs. **a** Dorsal view representation of *C. hirundinis* (1), laboratory-reared *C. lectularius* (2) and *C. hemipterus* (3). **b** Pronotum of *C. hirundinis* (**b**), *C. lectularius* (**c**) and *C. hemipterus* (**d**). Pronotal bristles and pronotum of *C. hemipterus* (**e** and** f**), *C. lectularius* (**g** and** h**) and *C. hirundinis* (**i** and** j**)

In total, 550 bugs were picked out from abandoned nests: 377 adults and 173 nymphs. Adult specimens were morphologically identified as *C. hirundinis*. They were characterized by the presence of long, pale bristles and less protruding eyes (Fig. 2a and b). Compared to our laboratory-reared bed bugs, they were smaller and more pubescent (Fig. 2a), and the anterior lobes of the pronotum (Fig. 2b) were moderately developed compared to the bed bug pronotums (Fig. 2c and d). The scanning electron microscope analysis revealed a detailed visualization of the species. The pronotum of *C. hirundinis* (Fig. 2j) compared to *C. lectularius* and *C. hemipterus* is remarkably less concave. At the pronotum sides, the bristles of *C. hirundinis* (Fig. 2i) are fine, longer and more numerous than the bed bug bristles (Fig. 2e and g), including on the whole-body surface (Fig. 2a and b). The *C. lectularius* pronotum bristle shape (forked-sharpened, showing jagged crowns) appeared to be identical to *C. hirundinis* pronotum bristles, but they were shorter and thicker (Fig. 2g and i). However, *C. hemipterus* bristles seemed to be smoother and not jagged (Fig. 2e). The *C. hirundinis* male intromittent genital organ is illustrated in Additional file 1: Figure S1a and c and the female paragenital sinus in Additional file 1: Figure S1b and d. Concerning the nymphs, we grouped the nymphs into stage 2, stage 3 and stage 4 based on body size (Additional file 2: Figure S2).

### Molecular identification of swallow bugs

**Fig. 3:**
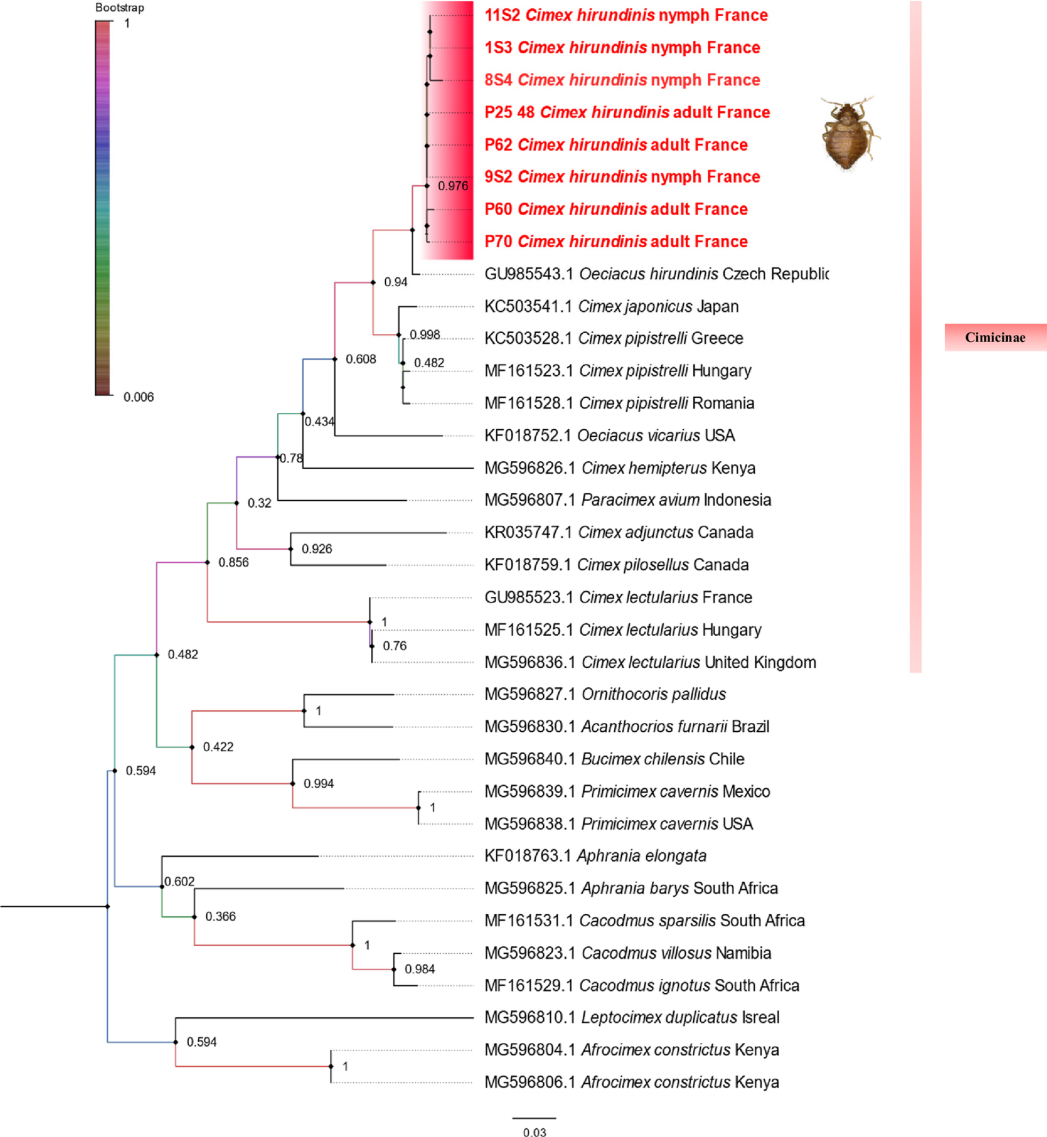
Phylogenetic tree based on *COI* gene sequences, including the sequences obtained from our study, showing the position of *C. hirundinis* (indicated in bold) compared to other species of different subfamilies. The colored vertical bar shows the sequences corresponding to the Cimicinae family. Although the *Oeciacus* spp. were recently shifted into the genus *Cimex* [39], the species nomenclature (*O. hirundinis*: GU985543.1) is provided as it remains in GenBank. The values on the branches are bootstrap support values based on 500 replications

The morphological identification of *C. hirundinis* was confirmed by molecular tools. Five sequences of adults and another four sequences of nymphs were successfully obtained using the *COI* gene. NCBI BLAST analysis of *COI* sequences from nine specimens of *C. hirundinis* added to our MALDI-TOF MS in-house database revealed that they were 98.66–99.12% identical to *Oeciacus hirundinis* (GenBank accession nos. MG596808-GU985544) (Table 2). To see the position of the obtained sequences among the GenBank *COI* sequences, a phylogenetic tree was constructed on the basis of *COI* fragment sequences. The phylogenetic tree showed that the sequence of *C. hirundinis* clustered with the sequence of *O. hirundinis* deposited in the GenBank and grouped in the Cimicinae subfamily (Fig. 3). The sequences of the *COI* gene of *C. hirundinis* were deposited in the GenBank. The FASTA of the *COI* sequence is attached in Additional file 3: Dataset S1.

### Comparison of *C. hirundinis* adults and nymphs

**Fig. 4:**
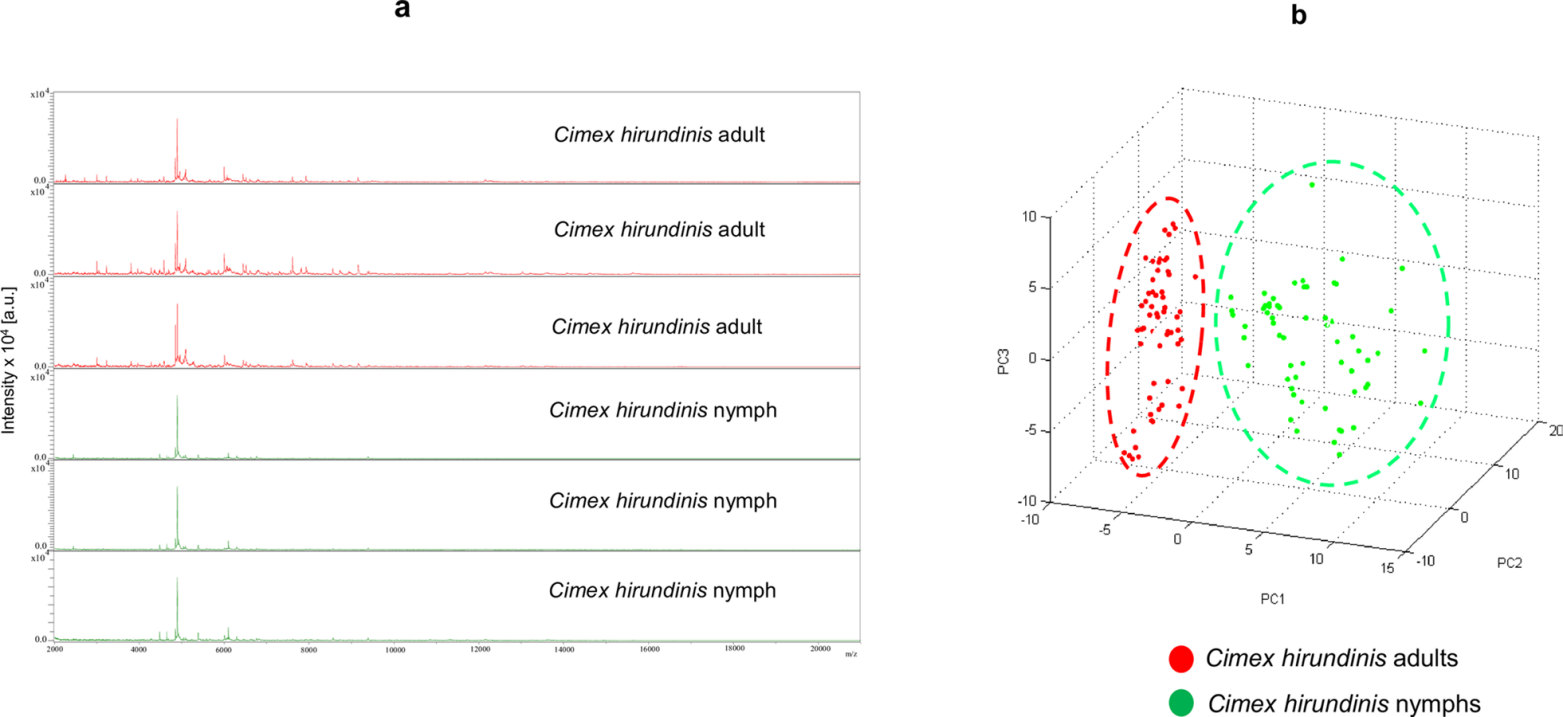
MALDI-TOF MS spectra obtained from *C. hirundinis* adults and nymphs: **a** Spectral alignment of MS spectra obtained from nymphs and adults using Flex Analysis software v.3.3. **b** Comparison of MALDI-TOF MS spectra of nymphs and adults on principal component analysis using ClinProTools v.2.2

**Fig. 5:**
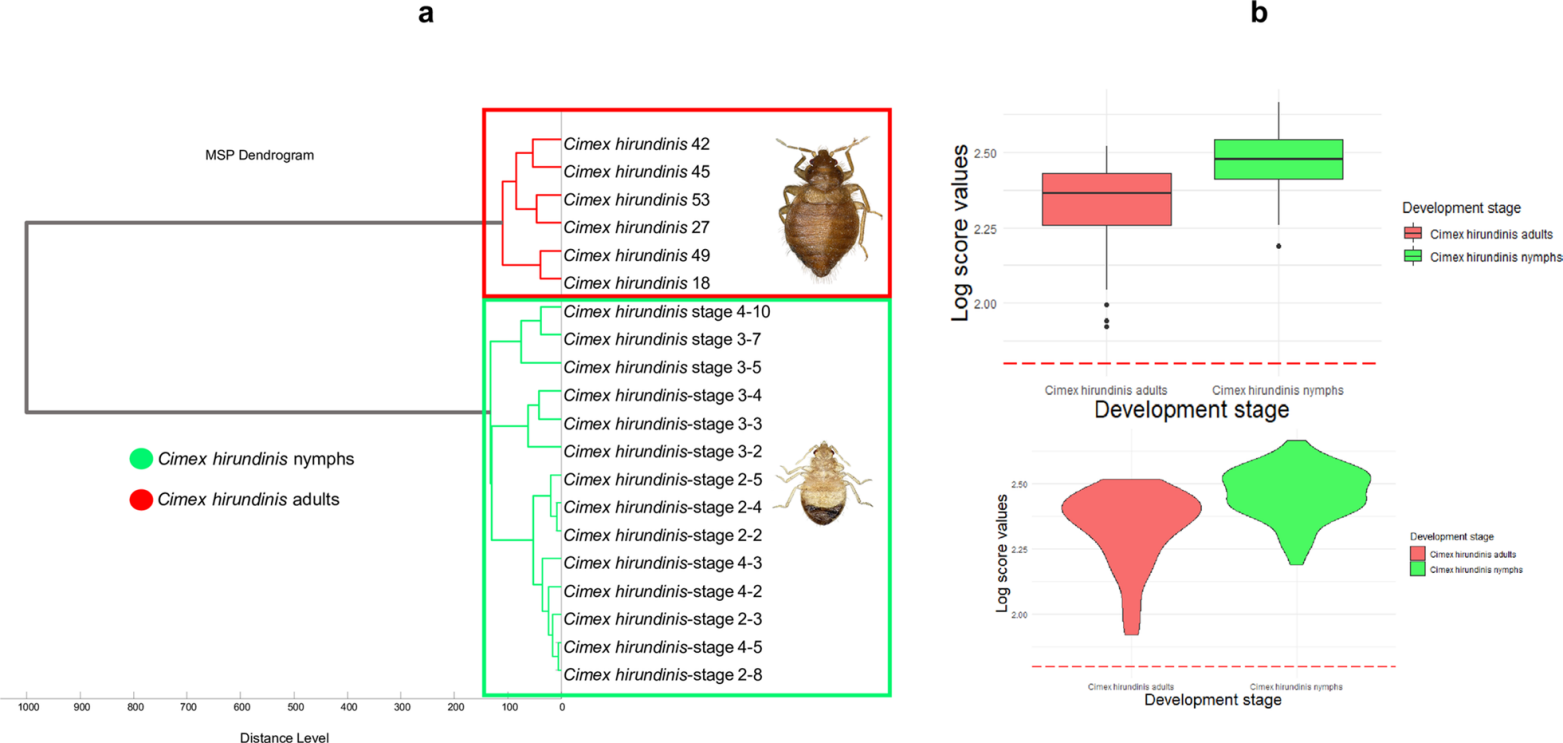
MS spectra obtained from adult and nymph specimens of *C. hirundinis*. **a** Hierarchical clustering dendrogram created using MS spectra from adult *C. hirundinis* and their nymphs. Cluster analysis was performed using Biotyper software v.3.0. **b** Graphical representation showing the distribution of log-score values according to the *C. hirundinis* developmental stages

Sixty-five specimens of adults and 50 nymphs (stage 2, 3 and 4), randomly selected, were subjected to MALDI-TOF MS to identify the specimens and assess the reproducibility as well as the specificity of MS spectra. Spectral profile analysis using Flex analysis software showed that 62/65 (95.38%) of *C. hirundinis* at the adult stage and 50/50 (100%) nymphs provided good-quality MS spectra (Table 2) (Fig. 4a). The principal component analysis using ClinProTools v.2.2 software revealed a clear distinction between nymphal and adult stages (Fig. 4b). The results were confirmed by the MS dendrogram (Fig. 5a) using MALDI-Biotyper 3.0. The MS protein profiles of both stages revealed sufficient discrimination between the MS spectra of adults and nymphs. However, clustering was not obtained for the nymphal stages (Fig. 5a).

**Table 2:** MALDI-TOF MS identification of *C. hirundinis* adult specimens and their nymphs

Developmental stage	Morpho ID	Specimens	Preservation method	No. tested	Good quality spectra	Molecular identification *COI* sequences	Reference spectra	Blind test	Bug species ID by MS	Score range
Nymphal Stage	*C. hirundinis*	173	− 20 °C	50	50/50	*O. hirundinis* (4) 98.94–99.12%	4	46/46	*C. hirundinis*	2.188–2.665
Adult stage		377		65	62/65	*O. hirundinis* (5) 98.66–99%	5	57/57	*C. hirundinis*	1.922–2.518
Total	–	550		115	112/115	9	9	103/103	–	–

### MS identification of *C. hirundinis* adults and nymphs

To validate the species identification by MALDI-TOF MS, five high-quality spectra of the adult stage and four spectra of the nymphal stage were randomly selected for database creation using MALDI-Biotyper 3.0 (Table 2). A blind test against our MS in-house database was carried out using the remaining spectra. The results showed that 100% (57/57) adult stage specimens were correctly identified with LSVs between 1.922 and 2.518. *Cimex hirundinis* nymphs were accurately identified, 100% (46/46), at the species level with LSVs between 2.188 and 2.665 (Table 2). All log-score values obtained for adults and nymphs are represented in Fig. 5b. For nymphs, the LSV mean was 2.461 ± 0.100 and the median was 2.476. For adults, the LSV mean was 2.328 ± 0.143 and the median was 2.363.

### Microorganism screening

**Fig. 6:**
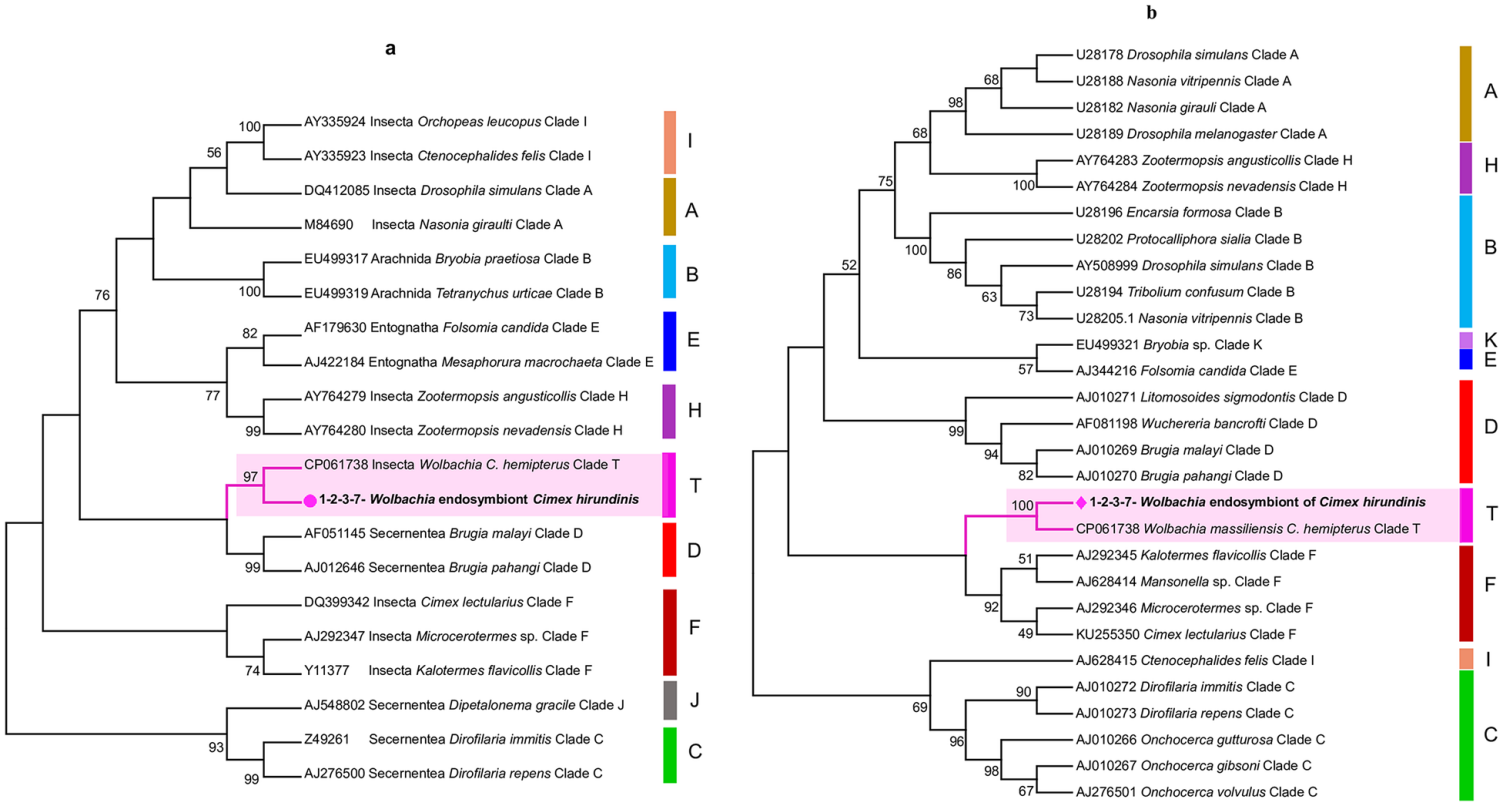
Phylogenetic analyses by the maximum likelihood method (the taxon in bold represents the *Wolbachia* of *C. hirundinis* and the letters indicate the *Wolbachia* supergroups). **a** *16S rRNA* gene phylogeny of various *Wolbachia* symbionts including *Wolbachia* from *C. hirundinis*. **b** Phylogenetic tree of *Wolbachia* symbionts based on *ftsZ* sequences, including the sequence obtained from *C. hirundinis*. The colored vertical bars indicate the *Wolbachia* supergroups. The values on the branches are bootstrap support values based on 1000 replications

One hundred fifteen specimens of *C. hirundinis*, including 65 adults and 50 nymphs, were first screened for the detection of microorganisms by qPCR. Of the *C. hirundinis* specimens, 99% (113/115) tested positive for *Wolbachia* spp. (*23S*) (Table 3). Four sequences of *16S* obtained from specimens positive for *Wolbachia* were identical to each other and showed 100% homology with the sequence of *Wolbachia massiliensis* isolated in *C. hemipterus* collected in Senegal (GenBank accession no. CP061738). Similarly, the NCBI BLAST analysis of *ftsZ* sequences revealed that the sequences were 100% identical to the sequence of *W. massiliensis* (GenBank accession no. CP061738) (Table 3). The phylogenetic analyses using the maximum likelihood method showed that the obtained sequences belonged to the new T supergroup and clustered with *W. massiliensis* isolated from *C. hemipterus* for both genes (Fig. 6a and b). No *Bartonella* spp., *Rickettsia* spp., *Borrelia* spp. and *C. burnetii* were detected.

**Table 3:** *Wolbachia* characterization based on *16S* and *ftsZ*

Morpho ID	Host	Country/province	No. of specimens positive for Anaplasmataceae spp. (%)	No. of specimens positive for *Wolbachia* spp. (%)	*16S rRNA*_%ID GenBank_Accession n = sequence	*ftsZ* gene_%ID GenBank_Accession n = sequence	Supergroup
*C. hirundinis*	*Delichon urbicum*	France/Toulouse	113/115 (99%)	113/115 (99%)	100% *W. massiliensis* (CP061738.1) (*n* = 4)	100% *W. massiliensis* (CP061738.1) (*n* = 4)	T

## Discussion

MALDI-TOF MS has increasingly been used in the clinical microbiology field for rapid and reliable microorganism classification, and its advantages are currently driving its application in routine microbiological laboratories [26]. More recently, this proteomic tool has also proven its effectiveness in malacology [40]. Several entomological researchers have reported the usefulness of MALDI-TOF MS as a time-saving, effective and less laborious approach for the identification of various arthropods (ticks, fleas, mosquitoes, bed bugs, biting midges, triatomines), targeting different body parts used for protein extraction that generate specific spectra for each species [25, 41–45].

The identification of species of the genus *Cimex* is complicated because it is based on different proportional measurements of the ratio between the length and the width of the pronotum and the length of bristles on the sides of the pronotum [6]. In addition, the use of this criterion alone is not enough to differentiate between species because of the closeness to the cutoff ratio of 2.5 [6, 46]. Therefore, in the present work, we showed the usefulness of MALDI-TOF MS as a complementary and alternative tool to rapidly identify swallow bug species (*C. hirundinis*) stored at − 20 °C, without requiring any entomological expertise. MALDI-TOF MS sample preparation is conditioned by different parameters (body part used, preservation method, extraction solution volume adjusted for protein extraction, homogenization method) that can affect the MS spectra quality [24]. *Cimex hirundinis* is the smallest species in Europe [7, 11]. Furthermore, if we compare this species (*C*. *hirundinis*) morphologically with the two bed bug species, it is noticeably smaller (Fig. 2a). Consequently, for the MS identification of nymphs, we selected the cephalothorax (head and thorax) as the body part and the extraction solution volume was adjusted to 15 µl, as the nymphs were smaller than adults. Moreover, we used glass beads as disruptors because they provided a simple, practical sample preparation and do not require any previous experience compared to glass powder. The current findings agree with previous studies on the ability of MALDI-TOF MS to distinguish between arthropod species [24, 27].

Based on the morphological criteria, the specimens collected from the house martin swallows were all identified as *C. hirundinis* at different stages, including adults and nymphs. In this study, we report the first case of swallow bug (*C. hirundinis*) invasion of a habitation in France (Fig. 1b–d). Hansel et al. [22] reported a similar case of human infestation by martin bugs (*C. hirundinis*) in Italy. Moreover, other human infestation cases with swallow bugs have been reported in the US and Japan [21, 47]. To date, in France, in addition to *C. hirundinis* we have found two species (*C. hemipterus* and *C. lectularius*) that bite and feed on human blood [5, 48, 49]. In the past, Lugger (1896) stated that bugs comparable to human bed bugs attacked swallows and bats. Those bugs were spotted in swallow nests, and they often reached human habitations, but morphologically the body was relatively smaller. Also, it has been previously reported that the American swallow bug (*C. vicarius*) was identical in general shape to the common bed bug (*C. lectularius*), but that swallow bug was smaller and had more bristles [50]. In the US, in the 1890s and 1900s, swallow bugs seem to have infested human habitations and been misidentified as bed bugs [4, 51]. Currently, most people would find it difficult to differentiate between *C. lectularius* and *C. hemipterus* [52]. Even if positively identifying bed bug infestation, they probably would not be able to discriminate the species. Consequently, when a bed bug infestation is considered, it is crucial to examine and identify the species, because in some cases the infestation might be due to either swallow bugs or bat bugs [22, 53]. In such cases, the application of the MALDI TOF MS approach is very interesting and recommended because it allows rapid and specific identification of the bugs, particularly when morphological identification becomes problematic for clinicians at the species level [23]. As recently stated, bed bugs were probably either misidentified as cockroaches because of their small size or not considered as insects at all in their early stages [23]. Here, we highlight the advantage of the MALDI-TOF MS technique to circumvent the drawbacks of morphological identification.

The upgrade of our in-house database with MS reference spectra of the relative method resulted in 46/46 (100%) *C. hirundinis* nymphs and 57/57 (100%) *C. hirundinis* adult specimens (Table 2) correctly and reliably identified at the species level with LSV > 1.8. Nymph identification was based only on size, and we estimated three stages, stage 2, 3 and 4. The dendrogram of MS spectra confirmed that the clustering was not observed according to nymphal stages, and this is explained by the fact that the nymph sizes were so diverse. Consequently, we could not perform a robust interpretation of results, but this does not affect the reliability of this tool. However, further studies are necessary to precisely identify the five nymphal stages of laboratory-reared *C. lectularius* and *C. hemipterus*.

In the current study, we demonstrated the strength of the congruence among MALDI-TOF MS, morphological and molecular identification. There was no ambiguity in the identification at the species level, which shows that this proteomic tool is fully valid, in concordance with other previous studies [27]. The molecular data analysis was based on the *COI* gene. This marker is widely used for different taxonomic and phylogenetic questions within Cimicidae and in the genus *Cimex* [39, 54]. Balvin et al. [39] proposed the genus *Oeciacus* as a synonym of *Cimex* based on molecular data analyses. Like Schuh and Weirauch [55], we followed this proposition in our publication.

In the microorganism screening section of our work, no pathogens were detected. To the best of our knowledge, infectious agents have not yet been documented in *C. hirundinis*, and it has never been proposed as a vector for human pathogens. However, one report mentioned *C. hirundinis* as a potential vector of paramyxovirus type 4 (0.1% infection rate in adult bugs and 0.4% in second to fifth nymphal stages, showing transstadial transmission) [56]. On the other hand, some arboviruses have been isolated from *C. vicarius*, emphasizing its vectorial role in transmitting the Buggy Creek virus, which causes western equine encephalitis. This raises a question about possible transmission to humans and livestock [13, 57, 58].

We detected a novel endosymbiont, *Wolbachia*, previously reported as *W. massiliensis* in the *C. hirundinis* studied here. Recently, this new strain was first isolated in *C. hemipterus* collected from Senegal and then described as a new clade (Clade T) [59]. In our study, we report for the first time to our knowledge a novel *Wolbachia* in the genus *Cimex*, specifically in the European swallow bug (*C. hirundinis*), of which the sequences had 100% homology with both *16S* and *ftsZ* sequences belonging to the T-supergroup strain (*W. massiliensis*). Based on previous studies, the infection of *Wolbachia* species of the F clade was common in the Cimicinae subfamily, and infection in the A clade is prevalent in the Afrocimicinae and Haematosiphoninae subfamilies [60]. At present, T-supergroup infections are introduced in two different species, *C. hirundinis* and *C. hemipterus*, which belong to the Cimicidae subfamily and originated from two different continents. Conversely, *Wolbachia* in the American swallow bug (*C. vicarius*) is phylogenetically classified in the F-supergroup [61]. In addition, a detailed study on *Wolbachia* diversity in bed bugs (*C. lectularius*) collected from different locations in France as well as other studies reported that the *Wolbachia* strain detected in *C. lectularius* belonged to the F supergroup [60–62]. Also, our findings revealed that the prevalence of *Wolbachia* obtained in studied *C. hirundinis* was visibly higher than that reported in *C. lectularius* from France [62]. In our study, we report for the first time to our knowledge the phylogenetic characterization of *Wolbachia* infecting *C. hirundinis,* revealing its classification in a new, recently discovered supergroup (T supergroup) associated with *C. hemipterus*. Thus, we support the suggestion made by Ros et al. [63] on the possibility of discovering other supergroups that taxonomically enlarge *Wolbachia* diversity as various potential host species are examined and screening methods improve.

## Conclusion

In the present study, we report for the first time to our knowledge a case of human infestation by swallow bugs (*C. hirundinis*) in France. This raises awareness of a new infestation that may easily be mistaken for bed bug infestation. Accordingly, in bed bug control missions, it is recommended to identify bird nests in the buildings or the surroundings, and the bugs involved, to avoid infestation recurrences. In addition, we showed the usefulness and robustness of MALDI-TOF MS in the rapid identification of adults and nymphs of *C. hirundinis* specimens with minimal sample requirements. However, further studies are required to validate the reliability of the MALDI-TOF MS protocol for other Cimicidae species to be fully incorporated in diagnostic routines. In this work, we also seized the opportunity to phylogenetically characterize the novel *Wolbachia* strain (*W. massiliensis*) infecting *C. hirundinis* and compared it to other recognized *Wolbachia* clades obtained from different arthropods. It is necessary to note the importance of identifying *Wolbachia* diversity in certain Cimicids infesting humans for control purposes.

## Supplementary Information

**Additional file 1: Figure S1.** Digital microscope (DM) and scanning electron microscope images (SEM) showing a ventral view of *C. hirundinis*: male intromittent organ (**a** and **c**); female paragenital sinus (**b** and **d**).

**Additional file 2: Figure S2.** SEM showing a representation of an egg and nymphs at different stages of *C. hirundinis*. **a** egg, **b** nymph II, **c** nymph III, **d** nymph IV. The nymphal stages were identified based on body size.

**Additional file 3: Dataset S1.** *COI* sequences of *C. hirundinis* adults and nymphs.

**Additional file 4: Dataset S2.** *16S* and *ftsZ* sequences of *Wolbachia* isolated from *C. hirundinis*.

## Data Availability

*COI* gene sequences of *C. hirundinis*: OK077757, OK077758, OK077759, OK077760 and OK077761. All FASTA sequences for the *Wolbachia* isolated from *C. hirundinis* are available in Additional file 4: Dataset S2.

## Abbreviations

*COI*: Cytochrome c oxidase I
RNA: Ribonucleic acid
qPCR: Quantitative polymerase chain reaction
