# Supplementary figures and images for: Molecular and MALDI-TOF MS identification of swallow bugs Cimex hirundinis (Heteroptera: Cimicidae) and endosymbionts in France
Source: Parasit Vectors. 2021 Nov 27;14:587. doi: 10.1186/s13071-021-05073-x (PMC8627032; doi:10.1186/s13071-021-05073-x)

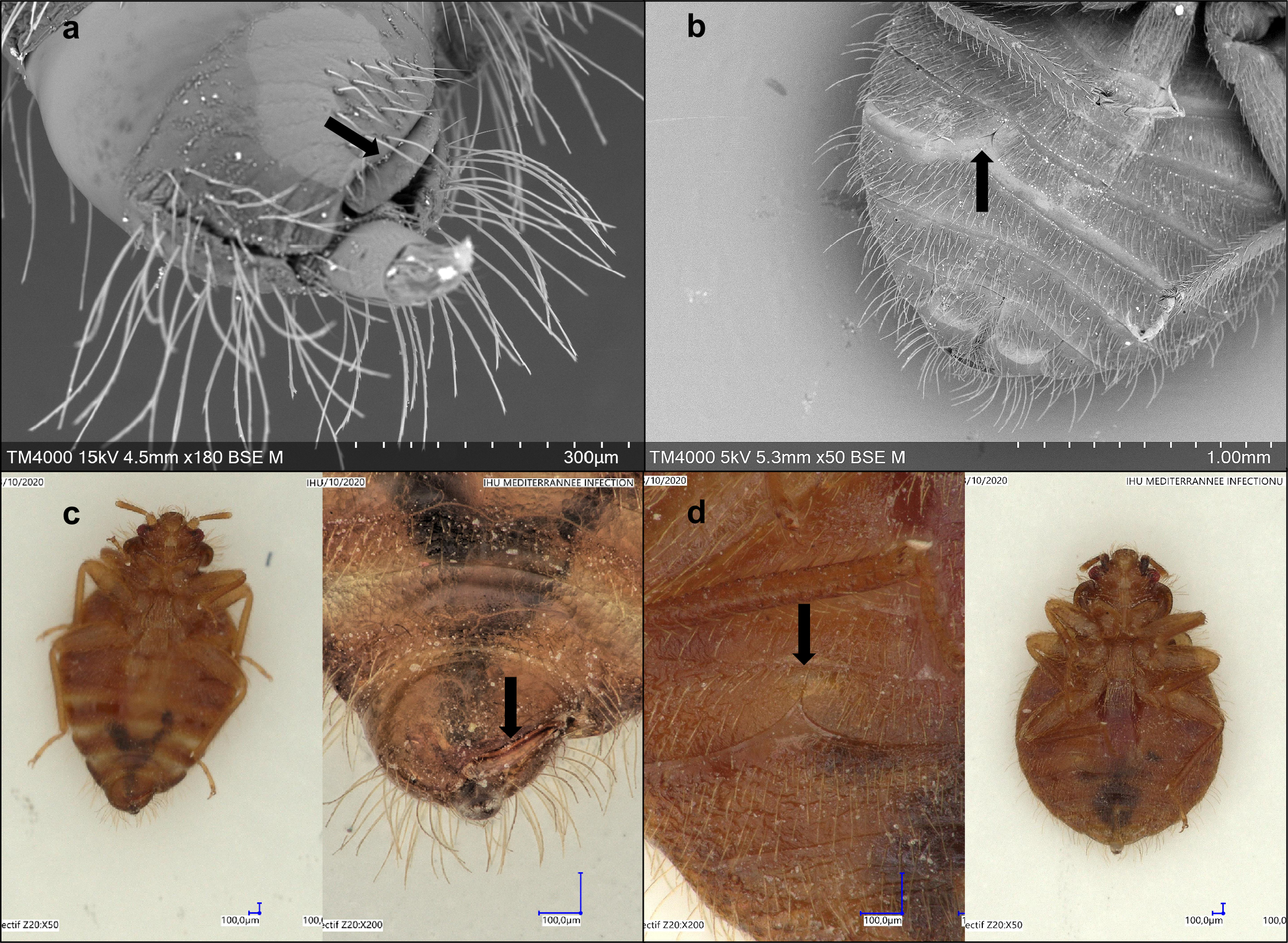

Supplement: Supplementary file 1 — Additional file 1: Figure S1. Digital microscope (DM) and scanning electron microscope images (SEM) showing a ventral view of C. hirundinis: male intromittent organ (a and c); female paragenital sinus (b and d). [file 13071_2021_5073_MOESM1_ESM.tif]

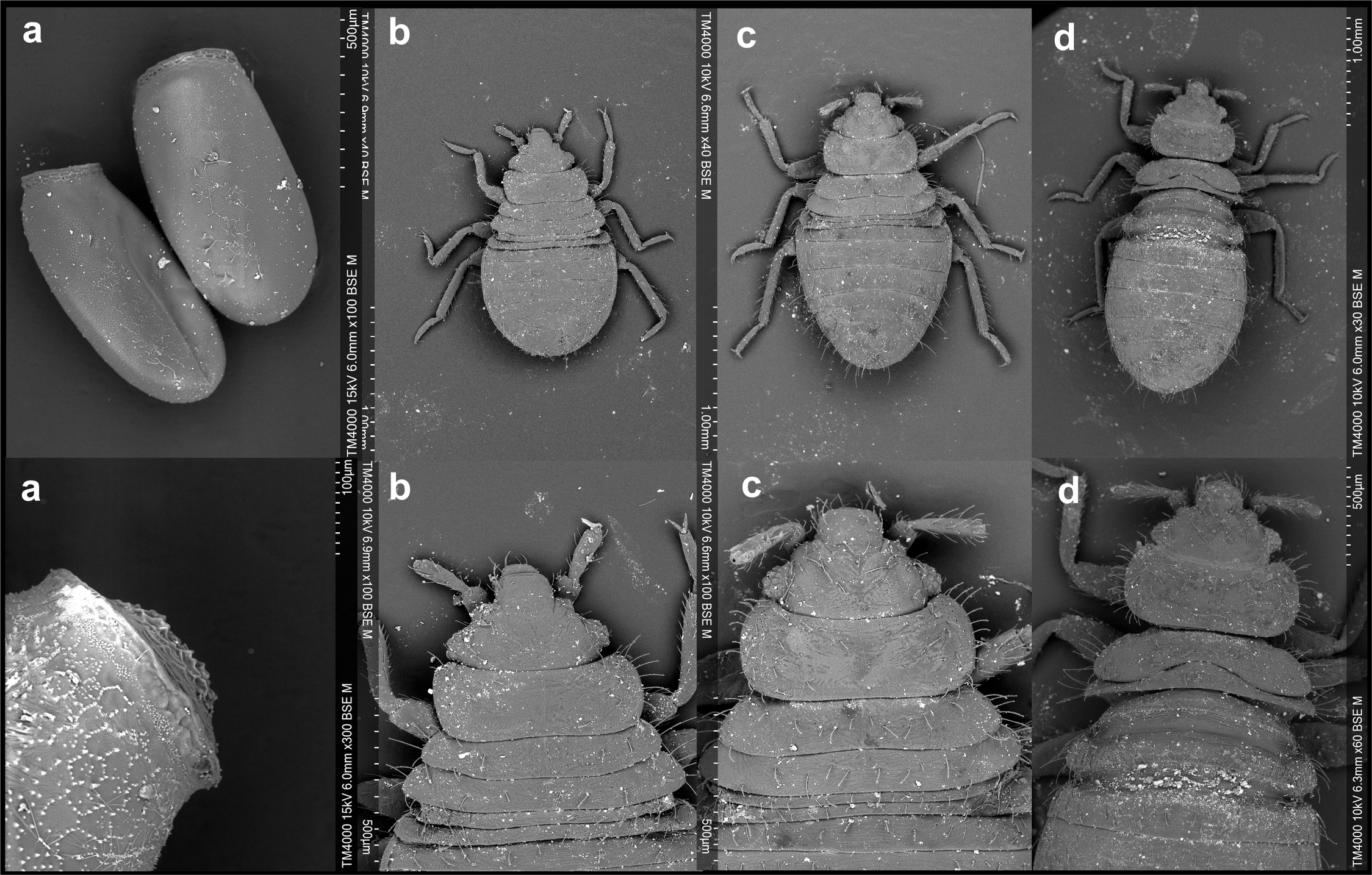

Supplement: Supplementary file 2 — Additional file 2: Figure S2. SEM showing a representation of an egg and nymphs at different stages of C. hirundinis. a egg, b nymph II, c nymph III, d nymph IV. The nymphal stages were identified based on body size. [file 13071_2021_5073_MOESM2_ESM.tif]
